# Duodenal Metastasis From Primary Lung Adenocarcinoma: A Diagnostic and Therapeutic Challenge

**DOI:** 10.7759/cureus.40821

**Published:** 2023-06-22

**Authors:** Philip Bouchette, Rachaita Lakra, Seth Haydel, Catherine T Hudson

**Affiliations:** 1 Internal Medicine, Louisiana State University Health Sciences Center, Shreveport, USA; 2 Internal Medicine, Leonard J. Chabert Medical Center, Houma, USA; 3 Gastroenterology, Louisiana State University Health New Orleans, New Orleans, USA

**Keywords:** immunohistochemical markers, esophagogastroduodenoscopy (egd), upper gastrointestinal(ugi) bleeding, duodenal metastasis, adenocarcinoma lung

## Abstract

Distant metastasis from primary lung cancer is mostly seen in the liver, brain, adrenal glands and bones. Small bowel, specifically duodenum is a relatively unusual site for distant metastasis from lung carcinoma. This case reports a rare scenario of upper gastrointestinal bleeding caused by duodenal metastasis by a primary lung adenocarcinoma. A 43-year-old woman presented to the emergency department with complaints of progressive hemoptysis for the past three weeks. Esophagogastroduodenoscopy (EGD) revealed a 2.5 cm x 2.5 cm fungating villous mass-like structure in the first portion of the duodenum, with a normal-appearing esophagus and stomach. Biopsies were performed, which were histologically consistent with poorly differentiated malignant. The immunohistochemical (IHC) staining was consistent with metastatic disease from primary lung adenocarcinoma. Due to its rarity, there are no solidified guidelines for the management of duodenal metastasis from lung carcinoma. Our case was challenging due to the extensive metastasis and low functional status of the patient and was ultimately managed with home hospice.

## Introduction

Lung adenocarcinoma (LADC) is a commonly diagnosed subtype of non-small cell lung cancer in patients globally constituting almost 40% of all such cases reported [1]. Even with substantial progress made in early identification and effective treatments today, LADC still continues to be a leading cause of death related to cancer owing to metastasis [2]. Metastasis usually occurs quite widely across multiple organs like the brain, and liver via lymph nodes amongst other organs; however small bowel involvement remains infrequent with only approximately 0.2%-1.7% incidence rates reported from primary lung cancers [3-4].

The pathophysiological mechanism that brings about GI metastasis post-LADC diagnosis remains poorly defined although it’s presumed by researchers that hematogenous or direct invasion routes could have a role to play here given the lack of satisfactory explanations thus far available [5]. As regards the exact molecular pathways involved such as angiogenesis and epithelial-mesenchymal transition, more research highlighting their significance during this process is still required [6]. Small bowel metastasis presents a clinical challenge due to its varying symptoms which often include abdominal pain, nausea, vomiting and GI bleeding [7]. Confirming a diagnosis therefore requires a combination of imaging studies and endoscopy evaluation as well as histopathological examination. Imaging modalities like computed tomography (CT) scans or magnetic resonance imaging (MRI), provide valuable information about potential lesions caused by malignancies while capsule endoscopy or double balloon enteroscopy facilitate direct visualization for biopsy in the small bowel [8].

## Case presentation

A 43-year-old woman, with a history of stage 4 adenocarcinoma lung, panlobular emphysema, latent tuberculosis, and 28 pack-per-year smoking history presented to the emergency department with complaints of progressive hemoptysis for the past three weeks. She described medium-sized blood clots during episodic coughing and occasionally hematemesis. The patient endorses overall worsening fatigue, myalgias, loss of appetite, and right hip pain.

The patient was diagnosed with metastatic lung adenocarcinoma, with 90% programmed death-1 ligand (PDL-1) positivity, one year ago and was managed with immunotherapy; pembrolizumab. She was also admitted at the hospital for pulmonary embolism a month ago and was presently on anticoagulation with apixaban. Patient reported non-compliance with her tuberculosis treatment.

On examination, the patient was afebrile, cachexic, tachycardiac to 114, and hypotensive, with a blood pressure of 96/57 mm hg. The patient was admitted to the intensive care unit for further management. Laboratory work was significant for hemoglobin (Hb) of 7.4 g/dL (patient’s baseline was 11-12 g/dL), alkaline phosphatase of 296 U/L, aspartate aminotransferase of 67 U/L, alanine transaminase of 56 U/L and lactic acid of 4.4 mmol/L. Other labs included a platelet count of 330 k/uL, prothrombin time of 12.4 sec, partial prothrombin time of 26.7 sec, international normalized ratio of 1.1, and total bilirubin 1 mg/dL (Table 1). She was initiated on proton pump inhibitors and received two units of packed red blood cells, with a repeat Hb of 9.9 g/dL.

**Table 1:** Laboratory Results g/dL=gram/deciliter; IU/L=international units per liter; U/L=units per liter; mmol/L=millimoles per liter; k/uL=kilo per microliter; INR=international normalized ratio; mg/dL=milligram/deciliter

Laboratory Test	Value	Reference Range
Hemoglobin	7.4	12.0-15.5 g/dL
Alkaline phosphatase	296	30-120 IU/L
Aspartate aminotransferase	67	8-33 U/L
Alanine transaminase	56	7-56 U/L
Lactic acid	4.4	0.5-2.2 mmol/L
Platelet count	330	150,000-450,000 k/uL
Prothrombin time	12.4	11-13.5 seconds
Partial prothrombin time	26.7	25-35 seconds
INR	1.1	0.8-1.1
Total bilirubin	1	0.3-1.9 mg/dL

**Figure 1:**
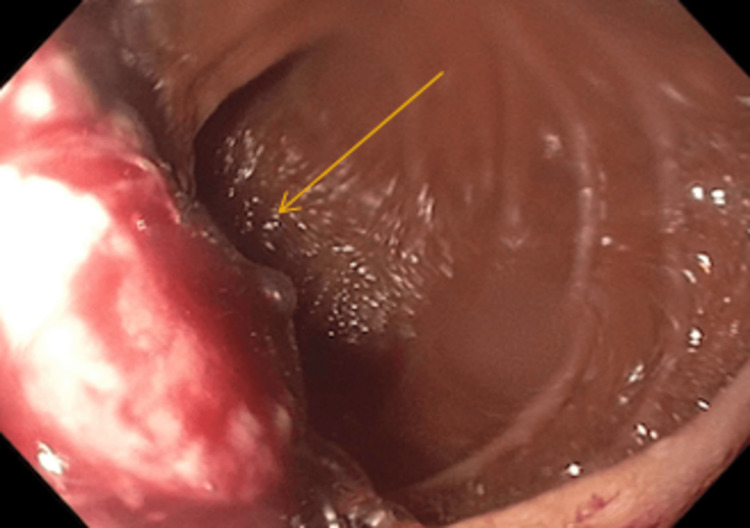
A medium-sized fungating villous mass-like structure with bleeding, in the first portion of the duodenum, with a normal-appearing esophagus and stomach.

The patient underwent an esophagogastroduodenoscopy (EGD), which revealed a 2.5 cm x 2.5 cm fungating villous mass-like structure in the first portion of the duodenum, with a normal-appearing esophagus and stomach (Figure 1). Biopsies were performed, which were histologically consistent with poorly differentiated malignant. The immunohistochemical (IHC) staining illustrated positivity for cytokeratin 7 (CK) and antibody cocktail AE1/AE3. IHC was negative for CK20, thyroid transcription factor 1 (TTF-1), and caudal-type homeobox 2 (CDX2) consistent with metastatic disease from primary lung adenocarcinoma.

**Figure 2:**
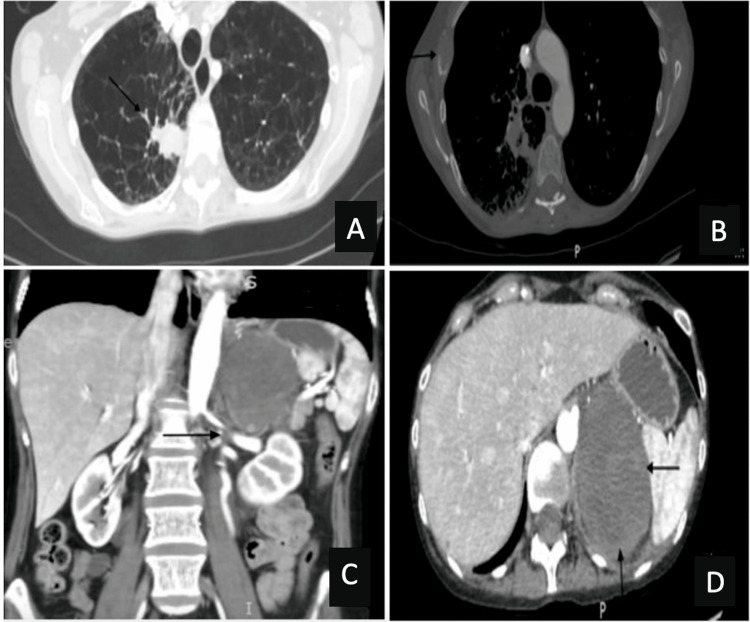
Computed tomography angiography (CTA) of the chest/abdomen and pelvis findings A. 2 cm area of consolidation in the posterior aspect of the right lower lobe in the axial plane. B. Lytic lesion involving the lateral aspect of the right fourth rib in the axial plane. C. Low-density filling defect suggestive of a thrombus in the left renal vein measuring 1.3 cm in the coronal plane. D. A large mass involving the left adrenal gland measuring 7.3 x 5.7 cm in greatest dimension which causes mass effect on the upper pole of the left kidney in the axial plane.

Computed tomography angiography (CTA) of the chest/abdomen and pelvis showed the previously visualized lung cancer with new lytic destructive lesions on multiple ribs. Additionally, there was a low-density filling defect suggestive of a thrombus in the left renal vein and a large mass involving the left adrenal gland which caused mass effect on the upper pole of the left kidney. All findings signifying worsening metastatic disease (Figure 2).

The patient’s Hb was stabilized with transfusion support and discontinuation of anticoagulation. Extensive multidisciplinary discussions were held with the patient and family, and the patient was discharged on home hospice.

## Discussion

Small bowel metastasis is often asymptomatic unless causing obstructive symptoms, which could contribute to its low incidence. Past literature reflects a low prevalence of GI involvement. Xu et al. [9] reported an incidence of GI metastasis from 366 cases of lung cancer as <2%, while Yang et al. [10] described a prevalence of 1.7%, whereas autopsy studies demonstrated a higher prevalence ranging between 4.7-14% which is significantly higher when compared to clinical statistics [11]. Jejunum (50.9%) is the most frequently involved site for small bowel metastasis from lung cancer, followed by ileum (33.3%) and rarely duodenum (15.8%) [12]. Our patient was diagnosed with adenocarcinoma lung, histologically, squamous cell carcinoma lung is more commonly seen as GI metastasis [13].

Presenting symptoms per previously reported cases included nausea, obstructive jaundice secondary to extraluminal compression of the biliary tree, small bowel obstruction, or perforation [14]. A case reported upper GI bleeding, due to metastatic involvement of the duodenal artery [15]. Our patient presented with signs of upper GI bleed caused by duodenal metastasis. The mode of lung cancer metastasis to the GI tract is attributed to both hematogenous and lymphatic spread but is not well understood [16]. Involvement of the GI tract could also indicate the presence of metastasis in other organs, which needs further imaging. Our patient was found to have new lesions in the adrenal glands, thyroid, and bones. A study reported that significant numbers of patients with small bowel metastases from primary lung cancer had more than one metastatic site and an average of 4.8 sites [17]. Another study recommended the consideration of GI metastases, upon involvement of abdominal lymph nodes and the adrenal glands [18].

CT scan evidence of GI involvement includes intraluminal mass, focal wall thickening due to infiltration, mucosal irregularities, or ulcerations [19]. A study reported 93% sensitivity of CT scans for detecting GI metastasis from lung cancer [20], our patient had no evidence of duodenal metastasis on CT scan. Positron emission tomography scans are also helpful in metastasis recognition but are not always feasible, like in the acute presentation of our patient.

Due to its rarity, there are no solidified guidelines for the management of duodenal metastasis from lung carcinoma. Treatment is patient-specific, endoscopic interventions for bleeding, palliative chemotherapy, or radiotherapy have been previously used with a median survival of 12-23 months [21]. Our case was challenging due to the extensive metastasis and low functional status of the patient and was ultimately managed with home hospice.

## Conclusions

Even though rare, GI metastasis from lung cancer cases is now more frequently reported, along with higher incidence of primary lung cancer itself. This case highlights duodenal involvement with adenocarcinoma lung, which is considered an infrequent metastatic site. Clinicians should consider GI metastasis on the differential upon encountering GI symptoms or anemia in a primary lung cancer patient.
